# The *Helicobacter pylori *duodenal ulcer promoting gene, *dupA *in China

**DOI:** 10.1186/1471-230X-8-49

**Published:** 2008-10-25

**Authors:** Zhiyu Zhang, Qing Zheng, Xiaoyu Chen, Shudong Xiao, Wenzhong Liu, Hong Lu

**Affiliations:** 1 Department of Gastroenterology, Shanghai Renji Hospital, Shanghai Jiaotong University School of Medicine, Shanghai Institute of Digestive Disease, Shanghai, PR China

## Abstract

**Background:**

The prevalence of *H. pylori *is as high as 60–70% in Chinese population. Although duodenal ulcer and gastric cancer are both caused by *H. pylori*, they are at opposite ends of the spectrum and as such are considered mutually exclusive. Duodenal ulcer promoting (*dupA*) gene was reported to be associated with duodenal ulcer development. The aim of this study was to determine the prevalence of *dupA *gene of *Helicobacter pylori *in patients with various gastroduodenal diseases and to explore the association between the gene and other virulence factors.

**Methods:**

*H. pylori *were isolated from gastric biopsies of patients with chronic gastritis, duodenal ulcer (DU), gastric ulcer (GU), or non-cardia gastric carcinoma. The *dupA*, *cagA*, *vacA*, *iceA *and *babA2 *genotypes were determined by polymerase chain reaction. Histological features of gastric mucosal biopsy specimens were graded based on the scoring system proposed by the updated Sydney system. IL-1β polymorphism was investigated using restriction fragment length polymorphism.

**Results:**

Isolates from 360 patients including 133 with chronic gastritis, 101 with DU, 47 with GU, and 79 with non-cardia gastric carcinoma were examined. The *dupA *gene was detected in 35.3% (127/360) and the prevalence DU patients was significantly greater than that in gastric cancer or GU patients (45.5% vs. 24.1% and 23.4%, *P *< 0.05). Patients infected with *dupA*-positive strains had higher scores for chronic inflammation compared to those with *dupA*-negative strains (2.36 vs. 2.24, p = 0.058). The presence of *dupA *was not associated with the *cagA*, *vacA, iceA *and *babA 2 *genotypes or with IL-1β polymorphisms.

**Conclusion:**

In China the prevalence of *dupA *gene was highest in DU and inversely related to GU and gastric cancer.

## Background

The morbility and mortality of gastric cancer rank the third in Chinese population and it accounts for around 0.3 million deaths per year. There is considerable interest in identifying virulence factors that are *Helicobacter pylori *disease specific (eg, related to duodenal ulcer and not gastric cancer). Several virulence factors such as the *cag *pathogenicity island, *vacA*, *oipA *and *babA *have been described and have been associated with an increase in the risk of both gastric cancer and duodenal ulcer disease [1-4]. They have also been associated with an increase in mucosal inflammation which is thought to underlie both duodenal ulcers and gastric cancer. Duodenal ulcer is associated with corpus sparing gastritis and gastric cancer with corpus atrophy and are clinically mutually exclusive diseases.

One problem that has possibly complicated identification of definite disease-specific *H. pylori *virulence factors is the considerable geographic diversity in the prevalence of *H. pylori *virulence factors. For example, in some regions, (ie, East Asia) the vast majority of strains have similar if not identical patterns of virulence factors such that potentially important factors can best be identified in regions where there is considerable diversity among strains. For example, the associations between the *cag *pathogenicity island, *vacA*, *oipA *and *babA *and enhanced mucosal inflammation, gastric cancer and peptic ulcer were identified and confirmed in Western countries where there is considerable strain diversity [5-9]. Polymorphism of interleukin-1β was reported to be an important host factor that increases the risk gastric cancer [10,11].

The duodenal ulcer promoting (*dupA*) gene was the first putative disease specific marker whose association was described using strains obtained from in both Asian (Japan and Korea) and Western (Colombia) regions [12]. *dupA *is though to be a *vir *homologue and the gene encompasses the sequences *jhp917 *and *jhp918 *as describe in strain J99. The original description of *dupA *reported that its presence was associated with increased mucosal neutrophil infiltration and its presence was inversely related to mucosal atrophy and gastric cancer. The aims of this study were to test the hypothesis regarding the association of *dupA *with the clinical outcome in a different population (ie, Chinese patients) as well as to test whether there were associations between *dupA *and previously described virulence factors or with proinflammatory interleukin-1β (IL-1β) polymorphisms.

## Methods

### Patients

Inclusion criteria included patients with documented *H. pylori *infection as evidence by positive *H. pylori *culture who underwent gastric endoscopy with biopsy specimens for *H. pylori *culture between January 2006 to August 2007 in the Department of Gastroenterology, Shanghai Renji Hospital, Shanghai, China. The patients involved were from 23 cities of China. All patients had simple *H. pylori *gastritis or a clinical *H. pylori*-related disease including: duodenal ulcer (DU), gastric ulcer (GU) or non-cardiac gastric adenocarcinoma. Simple *H. pylori *gastritis was defined as the presence of typical histological inflammation of gastric mucosa without peptic ulcer, gastric cancer or esophageal disease. Duodenal ulcers and gastric ulcers were identified endoscopically as active ulcers or ulcer scars. Exclusion criteria included negative results for culture, the presence of both duodenal and gastric ulcers or prior treatment for *H. pylori *infection. Patients with other primary malignancies, inflammatory diseases such as rheumatoid arthritis, or prior gastric surgery were also excluded. Written informed consent was obtained from all patients and the protocol was approved by the Institutional ethics committee of the Shanghai Renji Hospital based on the Helsinki Declaration.

### Biopsy protocol

Three biopsy specimens were taken from the greater curvature of the antrum in patients of gastritis, DU and GU. One specimen was used for *H. pylori *culture and two for histological examination. For gastric cancer and GU group one normal-appearing biopsy was taken culture and other 3 or 4 biopsies for diagnosis.

### *H. pylori *culture from biopsy specimens

The biopsies was inoculated onto brain heart infusion agar plates (Difco Laboratories, Detroit, USA) supplemented with 7% sheep blood, vancomycin (10 mg/mL), trimethoprim lactate (5 mg/L), amphoteracin-B (5 mg/mL) and of polymixin-B (2500 units/mL) and incubated in a microaerobic atmosphere (10%CO_2_, 85%N_2_, 5%O_2_) at 37°C for 5–7 days with 95% humidity. The organisms were identified as *H. pylori *by Gram staining, colony morphology and positive oxidase, catalase and urease reactions. Bacteria were sub-cultured using the same conditions.

### Histological evaluation

Gastric mucosal biopsy specimens were fixed in 10% buffered formalin, embedded in paraffin, cut in sequential 4-μm sections, stained with Haematoxylin & Eosin and modified Giemsa stain. One experienced pathologist blinded to the patient's clinical diagnosis examined the samples. Each specimen was scored for chronic inflammation, neutrophil infiltration, intestinal metaplasia and atrophy. Histological features were graded with the visual analog scale system graded from 0 (absent/normal) to 3 (maximal intensity) according to the scheme proposed by the updated Sydney system [13]. Each biopsy site was scored individually and the median score was determined for the all biopsy sites.

### DNA Extraction and PCR amplification

Bacterial chromosomal DNA was isolated from confluent plate cultures expanded from a single colony using the QIAamp Tissue kit (QIAGEN Inc. Santa Clarita, CA) according to the manufacturer's instructions. The isolated DNA was used as the template for PCR amplification. The 16S rRNA gene was amplified to confirm the presence of the isolated *H. pylori *strains. For analyses of the presence of target genes, *dupA*, *cagA, babA2, iceA *and *vacA *genotypes, *H. pylori *DNA were amplified using specific oligonucleotide primers described previously [Table 1] [14-18]. Primers of *jhp0917 *yielded a fragment of approximately 307 bp and primers of *jhp0918 *yielded a fragment of approximately 276 bp. PCR amplification was performed with a DNA Engine (MJ Research Inc., Watertown, Mass.) for 35 cycles consisting of 1 min at 95°C, 1 minute at 52°C and 1 minute at 72°C. The final cycle included a 7 min extension step to ensure full extension of the PCR products. The products of amplification were subsequently electrophoresed in 1.5% agarose gel stained with ethidium bromide to visualize the presence of amplified genes. *H. pylori *strain 26695 (ATCC700392) and J99 (ATCC700824) were used as negative and positive controls. The presence of *dupA *gene was defined as positive PCR results for both *jhp0917 *(product of 307 bp) and *jhp0918 *(276 bp product). If the PCR results yielded negative results, the isolate was considered negative for *dupA*.

**Table 1:** Primers used in the study

Gene	Primer Sequences	Reference
16S rRNA	5'-GCGCAATCAGCGTCAGGTAATG-3' 5'-GCTAAGAGATCAGCCTATGTCC-3'	14
cagA-3'region	5'-ACC CTA GTC GGT AAT GGG TTA-3' 5'-GTA ATT GTC TAG TTT CGC-3'	15
babA2	5'-AAT CCA AAA AGG AGA AAA AGT ATG AAA-3' 5'-TGT TAG TGA TTT CGG TGT AGG ACA-3'	17
iceA1	5'-GTG TTT TTA ACC AAA GTA TC-3' 5'-CTA TAG CCA STY TCT TTG CA-3'	18
iceA2	5'-GTT GGG TAT ATC ACA ATT TAT-3' 5'-TTR CCC TAT TTT CTA GTA GGT-3'	18
vacAs1a	5'-GTC AGC ATC ACA CCG CAA C-3' 5'-CTG CTT GAA TGC GCC AAA C-3'	16
vacAs1b	5'-AGC GCC ATA CCG CAA GAG-3' 5'-CTG CTT GAA TGC GCC AAA C-3'	16
vacAs2	5'-GCT AAC ACG CCA AAT GAT CC-3' 5'-CTG CTT GAA TGC GCC AAA C-3'	16
vacAm1	5'-GGT CAA AAT GCG GTC ATG G-3' 5'-CCA TTG GTA CCT GTA GAA AC-3'	16
vacAm2	5'-GGA GCC CCA GCA AAC ATT G-3' 5'-CAT AAC TAG CGC CTT GCA C-3'	16
jhp0917	5'-TGG TTT CTA CTG ACA GAG CGC-3' 5'-AAC ACG CTG ACA GGA CAA TCT CCC-3'	12
jhp0918	5'-CCT ATA TCG CTA ACG CGC TCG C-3' 5'-AAG CTG AAG CGT TTG TAA CG-3'	12

### IL-1β polymorphism

The genomic DNA was purified from 5 ml samples of peripheral bloods using Wizard Genomic DNA Purification kit (Promega) according to the manufacture's instruction. The polymorphisms (IL-1β-31 and IL-1β-511) were investigated using restriction fragment length polymorphism analysis of polymerase chain reaction products as previously studied [19]. PCR products were digested by restriction endonucleases (Alul for IL-1β-31 and Ava1 for IL-1β-511) and visualized by electrophoresis on a 2.5% agarose gel stained with 0.1% ethidium bromide.

### Data analysis

Chi-squire test and Fisher's exact test was used for univariate analysis. The significance of differences in histological features between *dupA *positive and negative groups was determined by comparing individual grades using the Mann-Whitney U test. *P *< 0.05 was taken to denote significance.

## Results

*H. pylori *isolates were obtained from 360 patients (235 men and 125 women; mean age of 53 years; range 17–90 years). The proportion of men was higher in the GU than in other three groups (p = 0.03) and the mean age of the patients with DU was lower than those with GU gastric cancer or gastritis (p = 0.03) (Table 2).

**Table 2:** Patients' characteristics in the study

	Men (%)	Mean age (range, years)
DU (n = 101)	59 (58%)	41 (17–72)
Gastritis (n = 133)	80 (61%)	59 (18–81)
Gastric cancer (n = 79)	54(68%)	59 (34–90)
GU (n = 47)	42 (89%)	58 (32–83)

### Detection of the *dupA *gene and clinical manifestations

**Figure 1:**
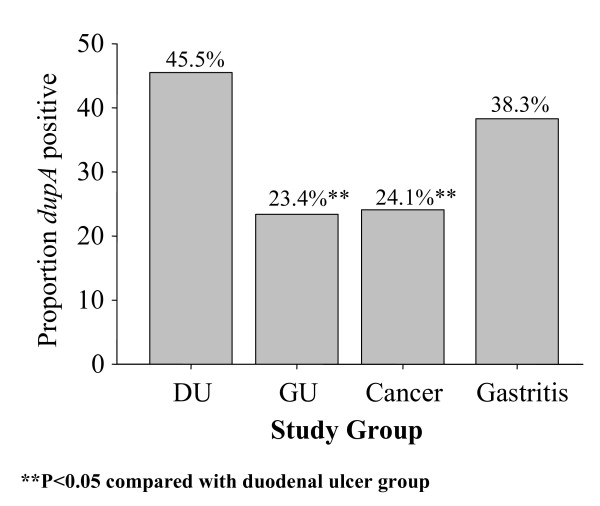
Prevalence of the *dupA *gene and clinical outcomes.

Overall, the *dupA *gene was present in 35.3% (127/360, 95% confidence interval (CI), 30.3–40.2%) of *H. pylori *strains isolated and the prevalence of the *dupA *gene was significantly higher in strains from DU (46/101, 45.5%, 95%CI, 38.2–55.2%) compared to those from gastric cancer (19/79, 24.1%, 95%CI, 14.6–33.5%) or GU (11/47, 23.4%, 95%CI, 11.3–35.5%) (*P *< 0.05 for both) confirming the original observation that *dupA *was related to DU and protective against gastric cancer (Figure 1). Fifty-one (38.3%, 95%CI, 30–45.6%) of the 133 patients with gastritis had *dupA-*positive strains which was higher than among those with gastric cancer but the difference did not reach statistically significance (*P *= 0.06). There is no significant difference between DU group and gastritis group (*P *= 0.2). The result also showed that the presence of the *jhp0917 *and *jhp0918 *genes was strongly linked (*P *< 0.001). Nine strains (2.5%) possessed *jhp0917 *positive/*jhp0918 *negative genotype and were classified as *dupA *negative. A *jhp0917 *negative/*jhp0918 *positive genotype strain was not detected.

### Association of *dupA *gene with histological findings

We compared the relationship between the present of *dupA *and the degree of chronic inflammation, neutrophil infiltration, atrophy and intestinal metaplasia in the antrum in the different groups except gastric cancer patients. Mann-Whitney U test showed that although patients infected with *dupA*-positive strains had higher scores for chronic inflammation compared to those with *dupA*-negative strains (2.36 vs. 2.24, p = 0.058), but the difference did not reach statistical significance (Table 3). The prevalence of the *dupA *gene was also independent of the scores of other histological variables including antral neutrophil infiltration, atrophy and intestinal metaplasia.

**Table 3:** Antral histological scores for the *dupA *matched groups

	Chronic inflammation	Acute inflammation	Atrophy	Intestinal metaplasia
dupA+				
Mean	2.36	1.31	0.74	0.49
Range	2–3	0–3	0–3	0–3
Median	2	1	1	0
dupA-				
Mean	2.24	1.3	0.63	0.47
Range	1–3	0–2	0–3	0–3
Median	2	1	0	0
Z	-1.92	-0.066	-0.956	-0.06
P	0.058	0.948	0.339	0.952

The individual grades for each histological feature were used in a Mann-Whitney U test to explore difference

### Association with other virulence factors

The only type of *vacA *signal sequence detected was s1a. Most (98%) strains were *cagA*-positive and 93% strains were *vacA *s1 genotype. The positive rate of *vacA m*1, *vacA m2, iceA1, iceA2 *and *babA 2 *of the 360 strains was 27%, 69%, 90%, 16%, and 64%, respectively (Table 4). The presence of *dupA *was not associated with any other virulence factors (P > 0.50 for all groups).

**Table 4:** Association between virulence factors and disease outcomes

Diseases	Patients No.	*dupA*	*cagA*	*vacA s1a*	*vacAm1*	*vacAm2*	*babA2*	*iceA1*	*iceA2*
DU	101	45.5%	98.0%	95.0%	18.0%	80.3%	60.6%	96.7%	19.8
Gastritis	133	38.3%	98.5%	90.9%	34.2%	64.3%	67.1%	88.6%	18.5
Gastric cancer	79	24.0%	97.5%	94.9%	34.2%	65.8%	68.4%	86.8%	21.0
GU	47	23.4%	97.8%	91.5%	20.0%	65.7%	60.0%	85.7%	14.2

### Association of *dupA *gene with IL-1β polymorphism

There were no significant differences in IL-1β genotype distribution between patients with *dupA *positive strains and those with negative strains (P = 0.50 for the patients with IL-1β-31 C carriers and P = 0.68 for IL-1β-511 T carriers).

## Discussion

Although DU and gastric cancer are both caused by *H. pylori*, they are at opposite ends of the spectrum and as such are considered mutually exclusive. DU is associated with sparing of the gastric corpus and high acid secretion whereas gastric cancer is associated with an atrophic pangastritis and low to absent acid secretion [20-22]. These different manifestations of the infection are thought to relate to as yet unexplained interactions between host and environmental factors and with bacteria virulence. Current virulence determinants including the *cag*-pathogenicity island, OipA, and BabA individually and together have been associated with an increased risk of ulcer or gastric cancer, however none has consistently shown specificity related to a specific pattern of gastritis or disease outcome.

The *dupA *gene is thought to be a homolog of the *virB4 *gene and is located in plasticity region of the *H. pylori *genome. Originally it was reported to be rare (9%) among patients with gastric cancer and common (42%) among patients with duodenal ulcer. As such, it appeared to be a marker for the presence of antral predominant gastritis and "protective" against the development of atrophic pangastritis. Using the same primers and primers of their own design, Arachchi *et al*. [23] confirmed that the *dupA *gene was present in approximately the same percentage of *H. pylori *strains isolated from DU patients (37%) in an Indian population as originally described [10]. They did not study patients with gastric cancer. A study in Brazilian adults [24] reported the *dupA *gene was present in 87% of patients with either DU or gastric cancer. They used their own primer set based on the sequences of Brazilian strains as well as the original primer sets. They subsequently reported identified two polymorphisms, an adenine deletion at the position 1311 and/or an adenine insertion after the position 1426 of the *dupA *gene in their isolates that led to different results [25]. They reported that the presence of wild *dupA *was significantly lower in gastric cancer (50%) than in gastritis (70%) or DU (78%). Finally, Argent *et al*. used the originally described primers and several other primer sets to examined *H. pylori *strains collected from Belgium (135 samples), South Africa (46 samples), China (31 samples) and the United States (46 samples) and reported that the prevalence of *dupA *gene was 50.6% of *H. pylori *strains isolated from DU patients and 71.1% from gastric cancer patients [26]. In this study, we evaluated Chinese isolates using the originally described primer sets. All patients had the typical East Asia type *H. pylori *genotype (ie, *cag*A+ve/*vacA s1*+ve) and *dupA *was present in 46% of strains from DU compared to 24% of patients with gastric cancer this confirming the original observations that *dupA *was commonly found in strains from patients with DU and infrequent among those with gastric cancer. The overall prevalence of *dupA *in Chinese isolates in study of Argent *et al*. was 32.3% which is similar to our result (35.3%). They only had one strain from a Chinese gastric cancer patient such that the DU: gastric cancer ratio could not be examined. In their study the results with China were lower than other three Western countries (43.5 to 84.8%). The difference between our studies and those of Argent *et al*. may be technical or relate to geographic variations circulation strains or difference in the definitions of patient groups [26]. In addition, we extended prior observations by showing that there was no relationship between *dupA *and previously proposed virulence factors (*cagA, vacA,, iceA *and *babA2*) or with host IL-1β polymorphisms.

Based on studies by the current author showing that presence of *dupA *appears to be associated with the absence of severe corpus gastritis or alternately with antral predominant gastritis, one can propose studies to directly test the hypothesis that *dupA *is associated with a particular pattern of gastritis. For example, the group of patients with *H. pylori *gastritis alone contains subgroups of patients some of whom will develop DU (ie, retain the antral predominant pattern), some destined to develop panatrophic gastritis and gastric cancer, and some who do neither. Thus, one would expect the presence of *dupA *to be inversely related to the severity of corpus gastritis. Unfortunately, the design of the current study did not allow us to test this hypothesis as we did not systematically collect corpus mucosal samples from the gastritis only group and we were only able to compare the severity of antral gastritis in relation to the presence of *dupA*. Patients infected with *dupA*-positive strains had higher scores for chronic inflammation compared to those with *dupA*-negative strains but the difference missed achieving statistical significance (p = 0.058). Initially *dupA *was identified in strain J99 where the gene was disrupted. Studies from Brazil have identified another truncation site [25] suggesting that PCR determination of functional *dupA *status may sometime provide misleading results. Interpretation of future studies would be improved if the presence of the DupA protein can be directly assessed as that would eliminate false positive PCR results which may as noted above fail to separate strains with a functionally inactive *dupA *from those that produce the DupA protein.

## Conclusion

Our present study showed that *dupA *gene was associated with DU in Chinese population, but its protective effects against atrophy/gastric cancer could not be confirmed. Similar to the other virulence factors of *H. pylori*, regional differences exist in the distribution of this gene.

## Competing interests

The authors declare that they have no competing interests.

## Authors' contributions

ZZ: cultured the bacteria and did genotyping, and reviewed the final manuscript. QZ: did genotyping, and reviewed the final manuscript. XC: checked the histological data, and reviewed the final manuscript. SX and WL: performed the gastroscopy, collected specimens, took care of patients involved, and revised the manuscript. HL: designed this study, analyzed the data, and has primary responsibility for writing the manuscript.

## Pre-publication history

The pre-publication history for this paper can be accessed here:


